# Effect of NO_2_ Aging on the Surface Structure and Thermal Stability of Silicone Rubber with Varying Al(OH)_3_ Contents

**DOI:** 10.3390/ma16062540

**Published:** 2023-03-22

**Authors:** Jiapeng Fang, Yi Luo, Shilong Kuang, Kai Luo, Zikang Xiao, Xiangyang Peng, Zhen Huang, Zheng Wang, Pengfei Fang

**Affiliations:** 1 Key Laboratory of Nuclear Solid State Physics Hubei Province, School of Physics and Technology, Wuhan University, Wuhan 430072, China; 2 Guangdong Key Laboratory of Electric Power Equipment Reliability, Electric Power Research Institute of Guangdong Power Grid Co., Ltd., Guangzhou 510080, China

**Keywords:** NO_2_ aging, SiR, thermal stability, surface structure

## Abstract

In this study, silicone rubber (SiR) with 0, 90, and 180 parts of aluminum hydroxide (Al(OH)_3_, ATH) contents prepared in the laboratory was treated in a certain concentration of NO_2_ for 0, 12, 24, and 36 h. Fourier transform-infrared spectroscopy (FT-IR), scanning electron microscopy (SEM), atomic force microscopy (AFM), X-ray photoelectron spectroscopy (XPS), and thermogravimetry (TG) were used to study the changes in the surface structure and thermal stability of SiR, as well as the influence of Al(OH)_3_ on the properties of SiR. According to AFM, the root-mean-square roughness of ATH-90 SiR was 192 nm, which was 2.7 times of ATH-0 SiR. With the incorporation of ATH, the surface of SiR became more susceptible to corrosion by NO_2_. According to FT-IR and XPS, with the increase in aging time, the side chain Si-CH_3_ of polydimethylsiloxane (PDMS) was oxidized gradually and a few of nitroso -NO_2_ groups were formed. According to TG, the incorporation of ATH caused the maximum decomposition rate temperature of PDMS to advance from 458.65 °C to 449.37 and 449.26 °C, which shows that the thermal stability of SiR degraded by adding ATH. After NO_2_ aging, a new decomposition stage appeared between 75 and 220 °C (stage Ⅰ), and this decomposition trend was similar to aluminum nitrate, which was proven to reduce the thermal stability of PDMS. The effects of NO_2_ on the surface structure and thermal stability of different ATH contents of silicone rubber were preliminarily clarified by a variety of characterization methods, which provided ideas for the development of silicone rubber resistant to NO_2_ aging.

## 1. Introduction

SiR (SIR) composite insulators have many advantages, such as light weight, strong mechanical property, good antifouling performance, special hydrophobic recovery and insulation, etc. [1,2,3,4,5], which can effectively reduce the occurrence of breakdown, pollution flashover [6,7], and other malignant accidents; therefore, silicon rubber has a wide range of applications in the field of high-voltage power transmission [8,9,10,11]. High temperature vulcanized (HTV) SiR is the main insulating material of composite insulators. HTV-SiR was prepared by adding polydimethylsiloxane (PDMS) as the main matrix, aluminum trihydroxide (Al(OH)_3_ denoted as ATH) as the flame retardant silica (SiO_2_) and reinforcing agent, and adding a small amount of other auxiliary reagents, such as vulcanizing agent, methyl silicone oil, etc. The main features of PDMS are the Si-O-Si long chain forms, the main skeleton that connects non-polar methyl groups with low surface energy, and the small molecule polymer on both sides of the Si-O main chain [12]. This structure can effectively shield the polarity of the Si-O bond. As a flame retardant in SiR, ATH has the following functions: When the local high temperature occurs, ATH is first decomposed to achieve the purpose of cooling [13]. Then, an inert barrier layer is formed at the ablative site and prevents the inward conduction of external heat. ATH decomposition not only directly affect the flame retardant and thermal stability of SiR, but also cause the increase in surface defects, resulting in water penetration, mechanical properties decline, and other phenomena [14,15,16].

As the service environment of composite insulators is complex, HTV inevitably presents different degrees of aging phenomena, including decreased hydrophobicity, insufficient mechanical strength, and decreased thermal stability [7,17,18]. According to the different influencing factors, HTV aging mainly includes physical aging, chemical aging, and discharge aging. At present, there are a large number of research works focusing on the effects of various aging factors on the properties of SiR.

Hakami [19] studied the effects of corona on SiR samples under various environmental conditions, including ultraviolet radiation and humidity. The relationship between the sealing performance and hardness and roughness of SiR materials after the thermal oxidation aging test was studied by Wu et al. [20]. Moreover, Bleszynski et al. [21] developed a model of oxidative aging, caused by the presence of electrolytic water salts, that leads to the formation of hypochlorous acid on energized high-voltage transmission lines in coastal environments. The results show that the strong oxidizing environment is highly destructive to the SiR polymer network, and is more destructive than the non-electrolytic standard water and salt solution at higher temperatures. Verma [22] applied multiple stresses (humidity, temperature, UV, and electrical stress) to HTV silicone-based rubber insulators to assess the long-term performance in different climatic conditions. Paul et al. [23] studied how SiR insulation changed when exposed to 500 kGy gamma rays, and the erosion resistance of the virgin and gamma-ray irradiated specimens has been classified using GoogLeNet convolution neural network. These works show that the effects of a variety of factors individually or comprehensively have been investigated on aging of composite insulators, but there are also some factors whose influence has not been thoroughly studied to date. NO_2_ is one of the important chemical aging factors [24,25,26], and the oxidation of NO_2_ is easy to change the chemical structure and physical properties of SiR, which leads to the deterioration of the entire composite insulator [27]. Therefore, the study of the influence of NO_2_ on SiR can further improve the understanding of the aging mechanism of external insulating SiR under service conditions.

The researchers also studied the effects of flame retardants and reinforcing agents on the properties of SiR materials [22,28]. Ullah et al. [29] investigated the multiple stress aging properties of HTV-SIR filled with different concentrations of silica and ATH under DC voltage for 5000 h, and found that the hybrid composite has strong aging resistance compared with pure HTV-SIR. Moreover, the authors [30] found that the addition of silica and nano/micro ATH fillers to SiR enhanced the thermal stability of the composites. Xue et al. [31] found that SiO_2_-filled SIR composites had lower thermal conductivity between 30 to 150 °C, and showed better resistance to arc aging due to their good thermal stability and thermal conductivity at high temperatures. Jeon et al. [32]. studied the influence of nano-ATH particles on the leakage marking resistance of silicon rubber, and found that with the increase in the concentration of nano-ATH filler, the thermal stability and leakage current characteristics were improved. This may be due to the fact that ATH/SIR nano-composite inhibited the thermal decomposition and surface charge transfer, in order to be consistent with the leakage marking resistance. Verma et al. [33] tested the long-term performance of SiR based polymer insulators under DC stress and controlled climate conditions. A DC stress study conducted for a long time shows that material degradation, depolymerization, and ATH loss can lead to low thermal stability, and accelerate thermal erosion and early failure.

The above results showed that SiR can produce a better flame retardant when the ATH content is higher, but beyond a certain range, it will affect the embodiment of other properties. However, it is still unknown how the ATH content influences the structures and properties of SiR after chemical aging, such as exposure to NO_2_. To explore the influence of ATH content on SiR in NO_2_ aging process, self-made SiR with different ATH contents was aged in NO_2_ for different times, and the surface properties and thermal stability were studied by Fourier transform-infrared spectrometer (FT-IR), thermogravimetry (TG) and scanning electron microscopy (SEM), which will help in predicting the influence of ATH in NO_2_ aging process.

## 2. Materials and Methods

### 2.1. Materials and Preparation

Vinyldimethyl-terminated raw SiR was obtained from Cixi Xin Rui Chemical Co., Ltd., China, whose CAS number is 53529-60-5. Silica (SiO_2_) was obtained from Guangzhou GBS High-Tech & Industry Co., Ltd., China, whose CAS number is 112945-52-5. Aluminum hydroxide (ATH) was obtained from Aluminum Corporation of China, Shandong, China, whose CAS number is 21645-51-2. The scale of ATH is 90 mesh grain size. Concentrated nitric acid with 65–68% mass fraction was supplied by Sinopharm Chemical Reagent Co., Ltd., Shanghai, China, whose CAS number is 7697-37-2. Copper sheet was obtained from Tianjin Kemiou Chemical Reagent Co., Ltd., Tianjin, China, whose CAS number is 7440-50-8.

The samples with different ATH contents used in this study were all self-made in the laboratory. The equipment included the mixer (Jinyinhe, NHZ-51, Foshan, China) and the vulcanizing machine (Jiangsu Tianhui, XLB-50, Jiangsu, China). The preparation process of SiR was as follows: First, according to the industry standard, the amount of raw SiR was 100 parts (100 g), and the amount of SiO_2_ was fixed at 25 parts (25 g), with a small amount of various organic additives in the mixer. When the mixture became a light blue colloid, 0, 90, and 180 parts (0, 90, 180 g) of ATH were mixed with the above substances and refined for 6 h. Then, by adding two parts (2 g) of curing agent, the solution was further mixed for 1 h below 75 °C. After evenly mixing the curing agent, it was poured into a mold size of 300 × 300 × 3 mm in the vulcanizing machine plate for vulcanization (temperature of 180 °C, pressure of 15 MPa, duration of 10 min). The second vulcanization was carried out in an electric blast oven (DZF-6030, Drying Equipment Factory Nanjing Jianpai, Nanjing, China) at 200 °C for 12 h. Finally, SiR samples with 0, 90, and 180 parts of ATH contents were obtained and labeled as ATH-0, ATH-90, and ATH-180, respectively

### 2.2. NO_2_ Aging Experiment

**Figure 1:**
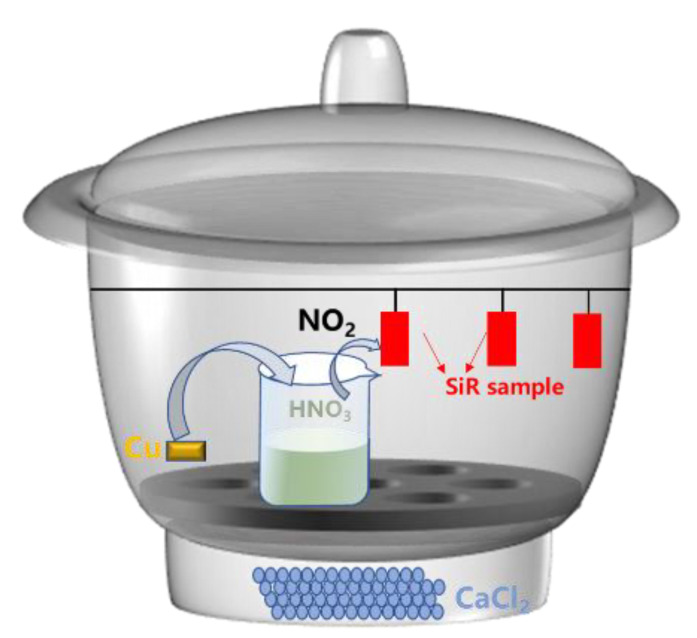
The schematic diagram of experimental device. NO_2_ was produced by the reaction of a copper sheet (Cu) with concentrated nitric acid (HNO_3_) and corroded silicone rubber (SiR) sample.

The schematic diagram of NO_2_ aging experimental device with volume of 2.5 L is shown in Figure 1. First, the researcher prepared four identical desiccators (Supin, Nantong, China), and then placed the excess anhydrous CaCl_2_ into the desiccants, which was dried in a sealed environment for 48 h. Second, four groups of SiR samples and four beakers with 20 mL (sufficient) of concentrated HNO_3_ were placed into each desiccator. Finally, four pieces of 0.01 g copper sheet were separately placed into the concentrated nitric acid to react and produce NO_2_. Simultaneously, all the dryers were sealed and saved in a drying oven (DZF-6030, Drying Equipment Factory Nanjing Jianpai, Nanjing, China) at a constant temperature of 25 °C. The experiment time of each test device was recorded respectively, in order that the samples were aged for 0, 12, 24, and 36 h in the atmosphere of a certain concentration of NO_2_, and then the aging samples were tested and analyzed.

### 2.3. Characterization

The chemical bonding states of SiR were analyzed by Fourier transform-infrared spectrometer-attenuated total reflection (FTIR-ATR) (Nicolet iS10, Thermo Electron Corporation, Waltham, MA, USA) and the wavelength range was 4000~400 cm^−1^. The morphologies of SiR were characterized by the field-emission scanning electron microscope (SEM, Hitachi S-4800, FEI Company, Hillsboro, OR, USA) equipped with an EDS detector, whose test conditions were as follows: Amplification factor of 1.00 k, length scale of 50 μm, working voltage of 2.0 kV, and working distance of 9.2 mm. The surface morphology of the SiR was characterized by atomic force microscope (AFM, Bruker Dimension Icon, Bremen, Germany). X-ray photoelectron spectroscopy (XPS, ESCALAB 250Xi, Thermo Fisher Scientific, Shanghai, China) was performed to analyze the element valence state of the SiR. The instrument for thermal analysis (TG) was Netzsch STA449 comprehensive thermal analyzer, Germany. The experimental parameters of thermal analyzer were as follows: The pressure value of the nitrogen cylinder was 0.01 MPa, and the pressure value of the air cylinder was 0.01 MPa. The corresponding flow rate of protective gas and purge gas was 10–20 and 10–20 mL/min, respectively. The baseline temperature range was 20–800 °C, in which the heating rate was 10 °C/min.

## 3. Results and Discussion

### 3.1. Surface Morphology of SiR before and after NO_2_ Aging

**Figure 2:**
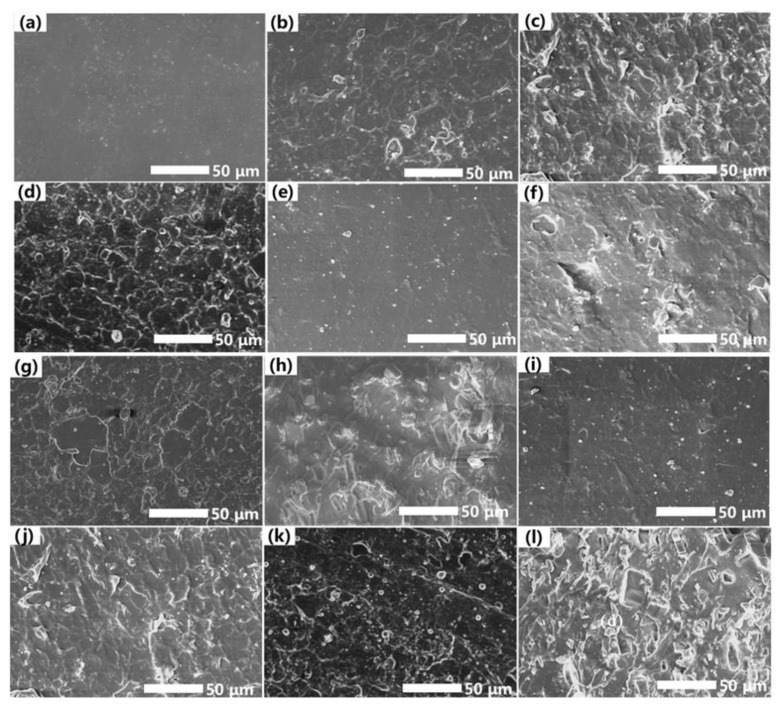
SEM images of ATH-0 SiR after NO_2_ aging for 0 h (**a**), 12 h (**b**), 24 h (**c**), and 36 h (**d**); SEM images of ATH-90 SiR after NO_2_ aging for 0 h (**e**), 12 h (**f**), 24 h (**g**), and 36 h (**h**); SEM images of ATH-180 SiR after NO_2_ aging for 0 h (**i**), 12 h (**j**), 24 h (**k**), and 36 h (**l**).

During the operation, an inorganic surface is an important feature of the aging of SiR composite insulator, which is generally manifested as the conversion of organic components to inorganic components and the exposure of inorganic fillers. The surface morphologies of ATH-0, ATH-90, and ATH-180 SiR after NO_2_ aging at different times (0, 12, 24, 36 h) were investigated by SEM in Figure 2. As shown in Figure 2a,e,i, the surface of SiR changed to a rough one with the incorporation of ATH. When the SiR surface is affected by NO_2_, the surface physical structure changes clearly. With the increase in aging time, the surface of the sample gradually exposed irregularly shaped particles and clear etching traces. It can be observed that a rougher surface of SiR will appear with the increase in ATH contents when corroded by NO_2_ for 36 h.

**Figure 3:**
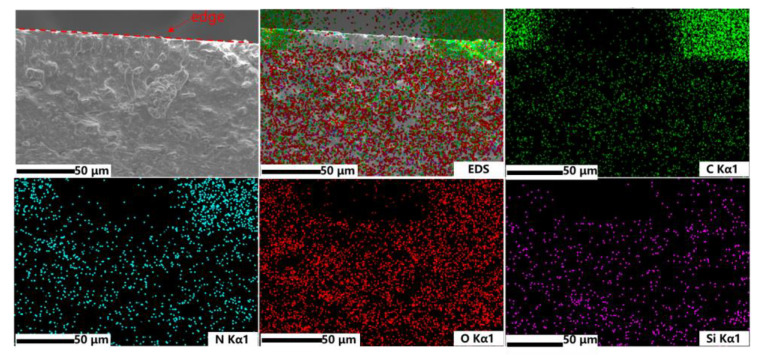
The EDS mapping result of C (green), N (blue), O (red), Al (purple) elements of cross-sectional ATH-90 SiR after NO_2_ aging for 36 h.

The EDS mapping spectra of C, N, O, Al elements of cross-sectional ATH-90 SiR were shown in Figure 3. The SEM image was the side section of the SiR after NO_2_ aging for 36 h, which appeared coarser than the unaged ATH-90 SiR (Figure 2e) and slightly smoother than the aged ATH-90 SiR (Figure 2h). In regard to the EDS spectrum of N element, the percentage composition of N was only 2.74 wt%, but it was distributed to almost 200 μm in depth under the surface of the sample. On this basis, it appeared that NO_2_ could infiltrate into the ATH-SiR for hundreds of μm.

**Figure 4:**
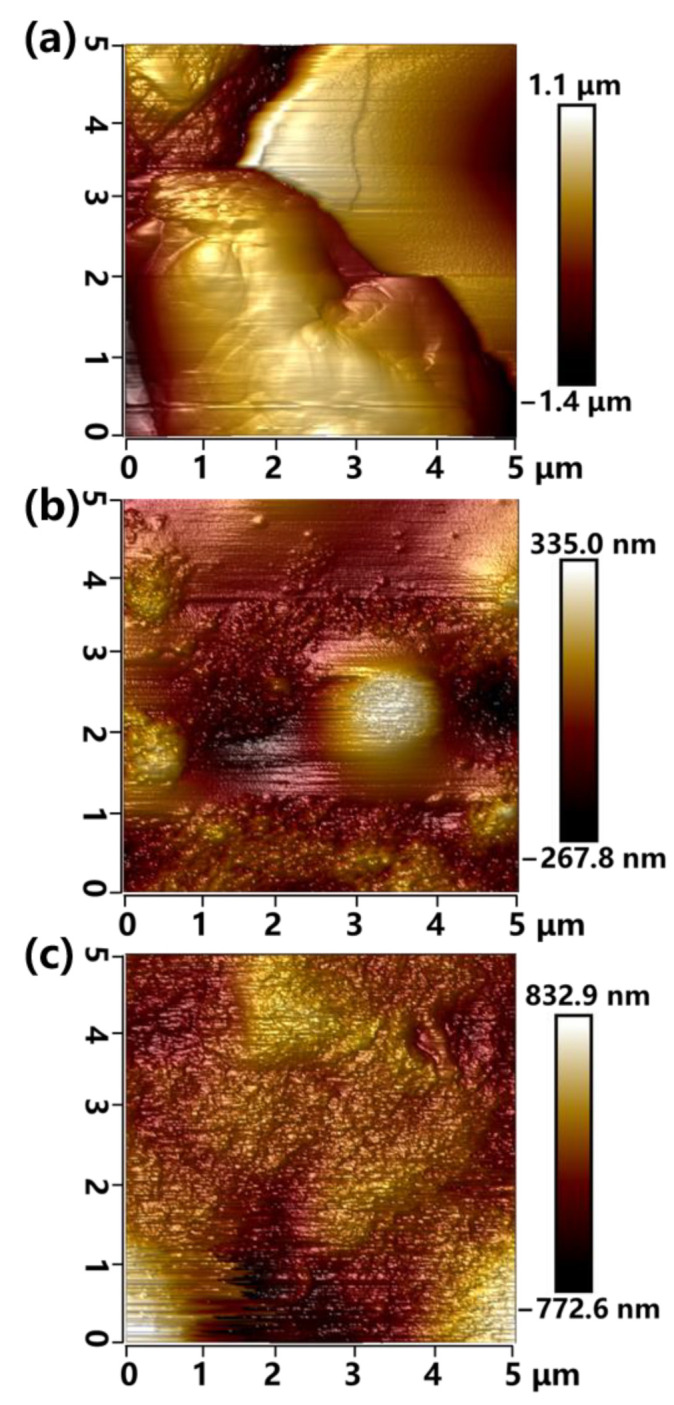
AFM 3D surface morphologies of ATH-90 SiR before NO_2_ aging (**a**), ATH-0 SiR after NO_2_ aging for 36 h (**b**), and ATH-0 SiR after NO_2_ aging for 36 h (**c**).

AFM experiments were used to examine the surface morphology of the SiR sample [34]. Figure 4a depicted the 3D surface height of ATH-90 SiR before NO_2_ corrosion. The surface revealed relative smoothness, except for the 2.5 μm height fluctuation which may have been caused by ATH contents. As shown in Figure 4b, the 3D surface of ATH-0 SiR after NO_2_ aging became rough and uneven, with many bumps compared with Figure 4a, which was the trace of NO_2_ etching. In addition, the root-mean-square roughness of ATH-0 SiR was 71.3 nm without ATH contents. As shown in Figure 4c, the root-mean-square roughness of ATH-90 SiR after NO_2_ aging increased once again since the root-mean-square roughness increased to 192 nm, which was 2.7 times of ATH-0 SiR. In addition, its surface had the most etching trace, which may indicate that with the incorporation of ATH, the surface of SiR became more susceptible to corrosion by NO_2_.

### 3.2. Surface Chemical Structure of SiR before and after NO_2_ Aging

**Figure 5:**
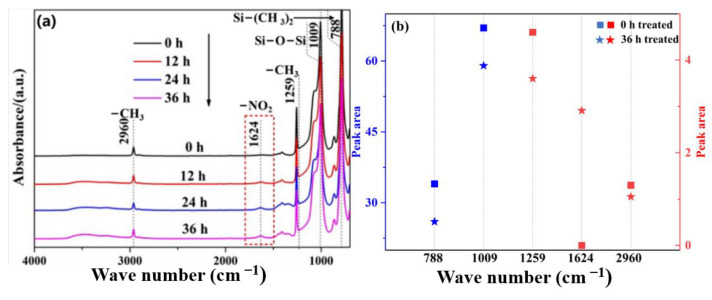
Infrared spectrum (**a**) and organic group peak area changes (**b**) in ATH-0 SiR after aging with NO_2_ for different times.

Figure 5a shows the corresponding infrared absorption spectra of ATH-0 SiR after aging for different times. Among them, 788 cm^−1^ is the stretching vibration peak of Si-(CH_3_)_2_, 1009 cm^−1^ is the stretching absorption peak of Si-O-Si, 1259 cm^−1^ is the symmetric deformation vibration peak of C-H in Si-CH_3_, and 2960 cm^−1^ is the asymmetric stretching vibration peak of C-H in -CH_3_ [27]. In addition, the asymmetric stretching vibration peak of -NO_2_ structure appeared in the aged samples at 1624 cm^−1^. Figure 5b shows a comparison of the peak areas at 0 and 36 h NO_2_ aging. With the increase in aging time, the -CH_3_ absorption peak at 2960, 1259, and 788 cm^−1^ weakens, while the -NO_2_ vibration peak at 1624 cm^−1^ gradually increases, indicating that NO_2_ will attack Si-C and C-H on the SiR side chain and introduce -NO_2_. In addition, the Si-O-Si absorption peak at 1009 cm^−1^ was weakened, indicating that the main chain of SiR (PDMS) was also broken under the action of NO_2_.

**Figure 6:**
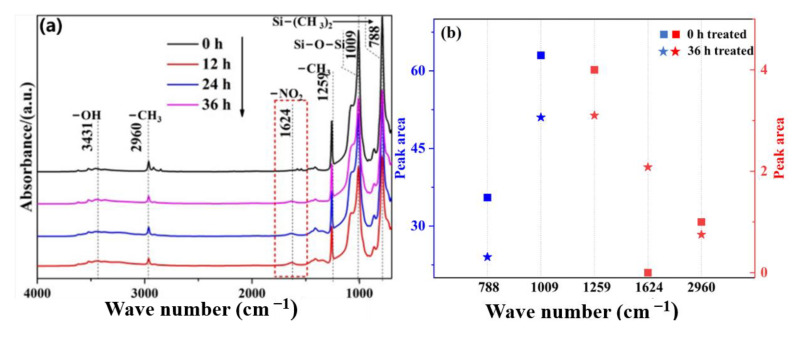
Infrared spectrum (**a**) and organic group peak area changes (**b**) in ATH-90 SiR after aging with NO_2_ for different times.

Figure 6 shows the corresponding infrared absorption spectra of ATH-90 SiR after aging for different times. Compared with ATH-0 SiR, ATH-OH stretched absorption peaks appeared at 3431 cm^−1^ in ATH-90 SiR. As can be seen from the figure, with the increase in aging time, the -CH_3_ absorption peak at 2960, 1259, and 788 cm^−1^ weakens, while the -NO_2_ vibration peak at 1624 cm^−1^ gradually increases, indicating that NO_2_ will attack the SiR side chain. The few hydrogen atoms on the side of methyl group were gradually replaced by the nitro group. When the aging time increased, the absorption peak of -OH slightly enhanced, which may be caused by the degradation of PDMS and the minor exposure of ATH.

**Figure 7:**
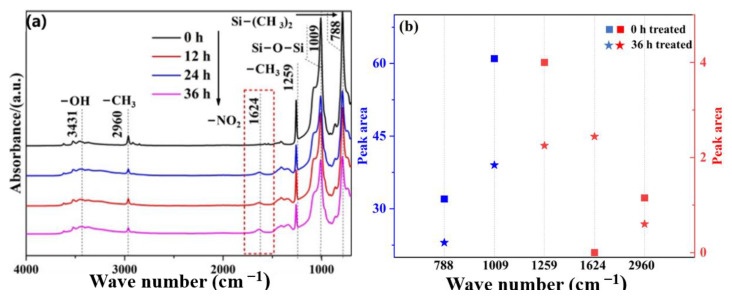
Infrared spectrum (**a**) and organic group peak area changes (**b**) in ATH-180 SiR after aging with NO_2_ for different times.

Figure 7a shows the corresponding infrared absorption spectra of ATH-180 SiR after aging for different times, whose characteristics were the same as those of ATH-90. Due to the addition of more ATH, a stronger stretching absorption peak of -OH appeared at 3431 cm^−1^. The increase in ATH reduces the relative content and strength of organic groups, such as -CH_3_ in SiR materials. Compared with ATH-90 SiR, the peak area of Si-O-Si and -CH_3_ is further decreased, which can be seen in Figure 7b, and the reduction amounts of organic groups increased. Moreover, this indicates that the increase in ATH will accelerate the aging process, which may be attributed to the effect of ATH on the physical structure of SiR, and thus accelerate the reaction of NO_2_ with the organic component of PDMS.

**Figure 8:**
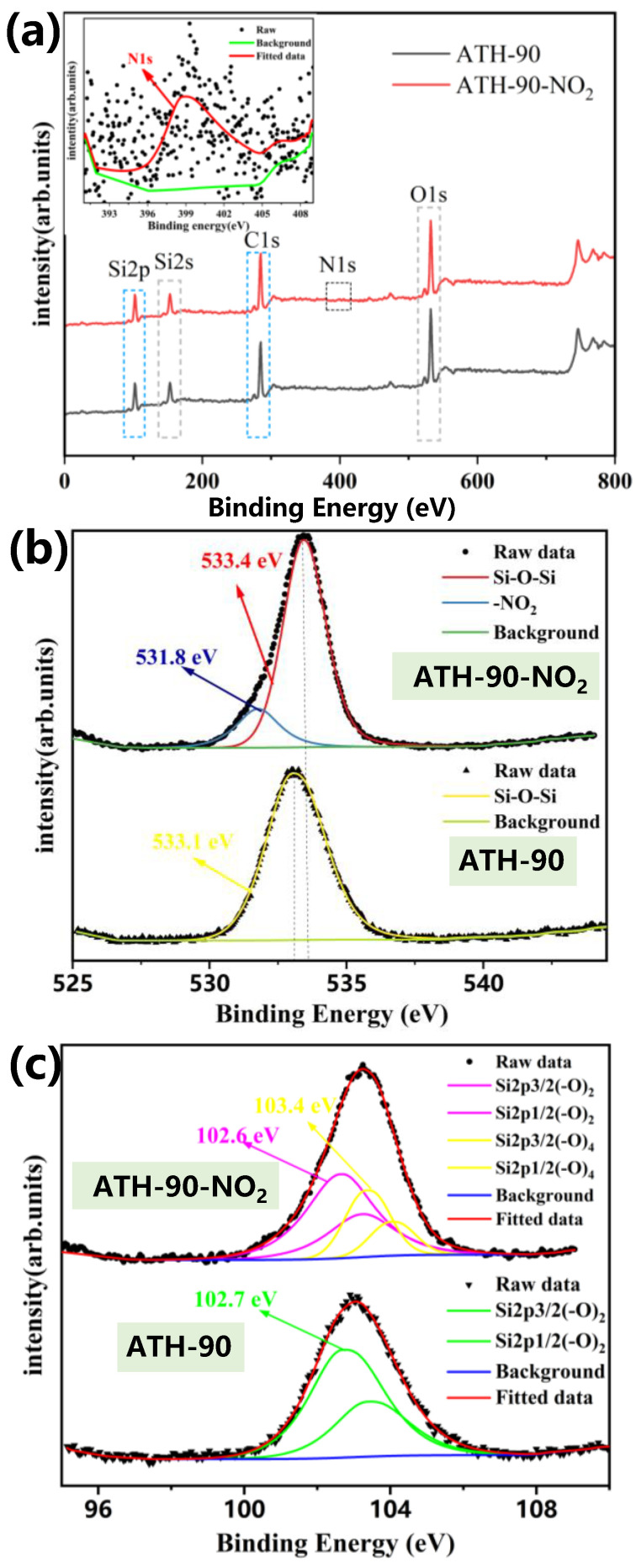
XPS analysis of survey (**a**), O 1s (**b**) and Si 2p (**c**) for ATH-90 SiR before and after NO_2_ aging.

Additional information regarding the elemental compositions and bonding characteristics of ATH-90 SiR before and after NO_2_ aging were analyzed by XPS (Figure 8). As can be seen by the XPS analysis of survey, the main peak respectively belonged to C 1s, O 1s, and Si 2p. There was a peak at 399.2 eV, which was corresponding to C-N, indicating that NO_2_ attacked SiR and produced -NO_2_ or -NH_2_ [35]. The O 1s XPS spectrum was shown in Figure 8b, wherein the peaks of ATH-90 SiR at 533.1 eV and ATH-90-NO_2_ at 533.4 eV were corresponding to Si-O-Si [35]. After NO_2_ aging, the spectrum of ATH-90 SiR markedly increased a peak at 531.8 eV. Combined with N 1s spectrum, it can be speculated that the surface of SiR had produced the NO_2_ group. The Si 2p XPS spectrum was shown in Figure 8c, wherein the peaks at 102.6 and 102.7 eV were corresponding to O-Si-O [36]. After NO_2_ aging, the spectrum of ATH-90 SiR markedly increased a peak at 103.4 eV, corresponding to Si-(O)_4_, which may be caused by the oxidation of PDMS side chains Si-CH_3_.

Therefore, combined with SEM, AFM, FT-IR, and XPS, the aging mechanism of NO_2_ was summarized as follows. First, NO_2_ attacked the side chains Si-CH_3_ of PDMS as well as produced the NO_2_ group. Second, the incorporation of ATH may have an impact on the physical structure of SiR, which will accelerate the aging process.

### 3.3. Thermal Stability of ATH SiR before and after NO_2_ Aging

**Figure 9:**
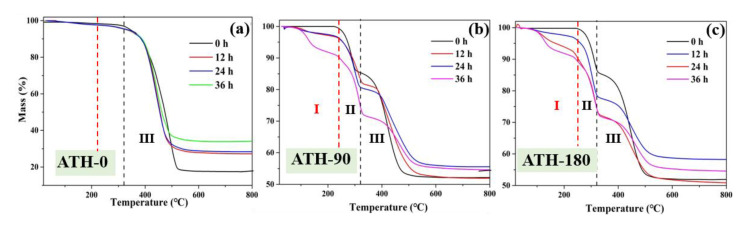
TG curves of ATH-0 SiR (**a**), ATH-90 SiR (**b**), and ATH-180 SiR (**c**) after NO_2_ aging for different times.

The thermal gravimetric (TG) curves of ATH-0 SiR were shown in Figure 9a. The mass loss between 320 and 600 °C (stage Ⅲ) was relatively clear, which was attributed to the decomposition of PDMS. In addition, the result has shown that the PDMS loss decreased gradually with the extension of aging time, which indicated that PDMS had been destroyed after NO_2_ aging. The effect of NO_2_ led to a certain reduction in the thermal stability of SiR. Compared with the SiR without ATH, it can be seen from Figure 9b that the new decomposition stage of ATH-90 without NO_2_ treated appeared at 220~320 °C (stage Ⅱ), which was attributed to the decomposition of ATH. After NO_2_ aging, a new decomposition stage appeared between 75 and 220 °C (stage Ⅰ), and it appeard that SiR had a new product. In addition, with the increase in ATH, the final mass loss of SiR, which was aged by NO_2_ for 36 h, increased gradually. Therefore, the incorporation of ATH and NO_2_ aging has a great influence on the thermal stability of SiR.

The mass loss of ATH-0 SiR, ATH-0 SiR, and ATH-0 SiR after NO_2_ aging for different times was listed in Table 1. In regard to ATH-0 SiR, the decomposition of small amounts of substances in stages Ⅰ and Ⅱ was attributed to the normal decomposition of other auxiliary fillers, which was not discussed here since the decomposition weight loss ratio was very small. Then, it was clear that the mass loss of stage Ⅲ decreased gradually for three groups of ATH SiR, with the increase in NO_2_ aging time. In regard to ATH-0 SiR, the PDMS mass loss of ATH-0 SiR decreased from 79.2% to 61.3% after the NO_2_ aging for 36 h, and the maximum advanced loss was 17.9%. However, in regard to stages Ⅰ and Ⅱ, the mass loss has shown a gradual increase over the NO_2_ aging time, which may hint that ATH SiR produces a by-product when treated by NO_2_.

**Figure 10:**
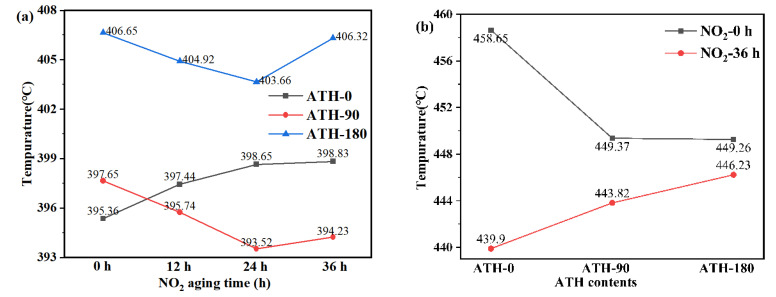
The initial decomposition temperature curves of PDMS (**a**) and the maximum decomposition rate temperature curves of PDMS (**b**).

The thermal properties of SiR were analyzed by initial decomposition temperature and maximum decomposition rate temperature. According to the International Thermal Analysis Association ICTA, the epitaxial initial decomposition temperature is usually used to express the initial decomposition temperature of the reaction. The initial epitaxial temperature refers to the intersection point between the tangent line of the steep part of the initial edge of the peak and the epitaxial baseline in thermogravimetric curve, while the point of maximum decomposition rate is the inflection point temperature, which corresponds to the peak point of the first-order differential curve. The changes in the initial decomposition temperature curves of PDMS were shown in Figure 10a. Before NO_2_ aged, the incorporation of ATH promoted the initial decomposition temperature of PDMS from 395.36 °C to 397.65 and 406.65 °C. On the contrary, the initial decomposition temperature of PDMS in ATH-90 SiR was lower than ATH-0 SiR, wherein it seemed that ATH can result in the premature decomposition of PDMS after NO_2_ aging. The curves of PDMS were shown in Figure 10b, wherein the incorporation of ATH caused the maximum decomposition rate temperature of PDMS to advance from 458.65 °C to 449.37 and 449.26 °C, which indicated that the thermal stability of SiR degraded after adding ATH. The incorporation of ATH may have an impact on the physical structure of SiR, which will accelerate the aging process. Similarly, the aging of NO_2_ could reduce the thermal stability of PDMS.

**Figure 11:**
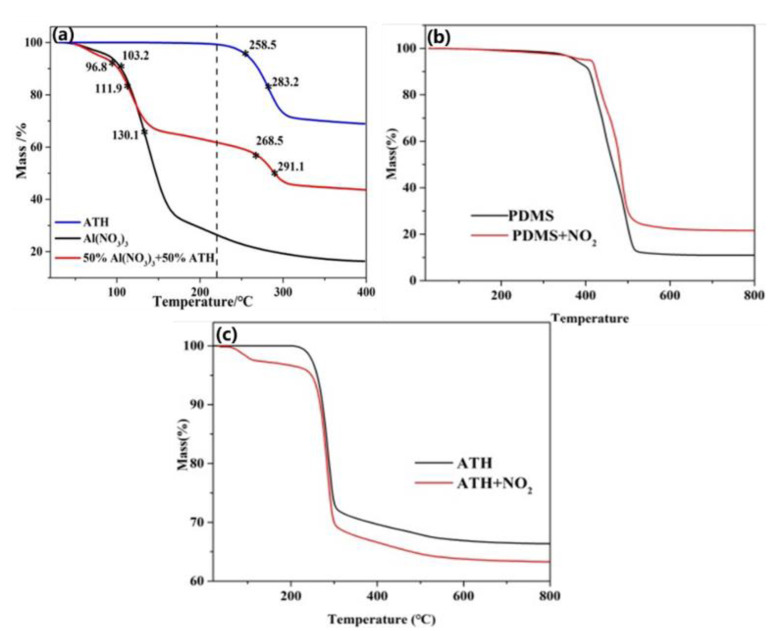
Thermogravimetric curve of ATH, Al(NO_3_)_3_ and their mixture (**a**), thermogravimetric curve of PDMS and PDMS aged for 36 h by NO_2_ (**b**), and thermogravimetric curve of ATH and ATH aged for 36 h by NO_2_ (**c**).

As seen in Figure 11, TG curves had a large fluctuation in stage Ⅰ. To explore the possible reasons for the differences, the mixtures of ATH and Al(NO_3_)_3_ as well as ATH and Al(NO_3_)_3_ were respectively tested by thermal analysis in this work. Figure 11a shows the TG curves of the above compounds and mixtures as well as the corresponding initial decomposition temperature and maximum decomposition rate temperature. It can be seen that the mass loss process of the mixture of ATH and Al(NO_3_)_3_ was concluded in two stages, while Al(NO_3_)_3_ or ATH only existed in one decomposition stage. The initial decomposition temperature and maximum decomposition rate of the mixture in the first stage were 96.8 and 111.9 °C, respectively, and the second stage corresponded to 268.5 and 291.1 °C. The initial decomposition temperature and maximum decomposition rate of Al(NO_3_)_3_ were 103.2 and 130.1 °C, respectively, and ATH were 258.5 and 283.1 °C, respectively. It can be found that when the temperature was lower than 220 °C, the mixed sample with Al(NO_3_)_3_ will decompose in advance compared with the single ATH, and the decomposition temperature will be around 100 °C. When the temperature was higher than 220 °C, the initial decomposition temperature and maximum decomposition rate of ATH corresponding to the mixture were increased by 10 and 8 °C compared with the elemental ATH.

Figure 11b,c shows the TG curves of pure PDMS and ATH after aging in NO_2_ for 36 h, respectively. It can be seen that the content of PDMS decreases due to the aging of NO_2_. This indicated that PDMS reacts with NO_2_, which was consistent with the results in AFM, FT-IR, and XPS. In regard to the TG changes before and after aging of ATH in Figure 11c, it can be seen that new mass loss occurred in the abovementioned stage Ⅰ, which was consistent with the aging characteristics of formed SiR, indicating that NO_2_ reacts with ATH and may generate Al(NO_3_)_3_ [27]. However, it is worth noting that the aging of NO_2_ increased the percentage of weightlessness corresponding to the main decomposition stage of ATH, mainly due to the fact that the relative molecular mass of Al(NO_3_)_3_ is greater than ATH, and the mass corresponding to the generation of the same amount of Al(NO_3_)_3_ was greater than the mass of ATH participating in the reaction. With the gradual increase in Al(NO_3_)_3_ generation, the weight loss ratio of SiR samples in the first stage increases gradually. Therefore, stages Ⅰ and Ⅱ corresponded to the mass loss of thermal decomposition of ATH and Al(NO_3_)_3_. This indicated that Al(NO_3_)_3_ may exist in the sample after NO_2_ aging, which reduced the thermal stability of ATH SiR.

## 4. Conclusions

In summary, combined with SEM, AFM, FT-IR, and XPS, the NO_2_ aging mechanism of surface structure was summarized as follows. First, NO_2_ attacked the side chains Si-CH_3_ of PDMS as well as produced the NO_2_ group, and the surface of SiR gradually exposed shaped particles and clear etching traces with the increase in NO_2_ aging time. In addition, NO_2_ could infiltrate into the ATH-SiR for hundreds of μm. Moreover, with the incorporation of ATH, the surface of SiR became more susceptible to corrosion by NO_2_. For thermal stability, PDMS loss decreased gradually with the extension of aging time since it had been distorted after NO_2_ aging. Furthermore, the thermal stability of SiR degraded when adding ATH. TG curves have shown that the incorporation of ATH will introduce a new decomposition stage, which was similar to Al(NO_3_)_3_, and the reason for the decrease in thermal stability of ATH SiR was due to the fact that the Al(NO_3_)_3_ may exist in the sample after NO_2_ aging.

The effects of NO_2_ on the surface structure and thermal stability of different ATH contents of silicone rubber were preliminarily clarified by a variety of characterization methods, which provided ideas for the development of silicone rubber resistant to NO_2_ aging. In the future, to improve the thermal stability of silicone rubber insulator, the researcher would like to seek a new nano-filler to improve the thermal stability of silicone rubber as well as the resistance to nitrogen dioxide corrosion.

## Figures and Tables

**Table 1:** The mass loss of ATH-0 SiR, ATH-0 SiR, and ATH-0 SiR.

	Mass Loss	ATH-0 SiR(%)		ATH-90 SiR		ATH-180 SiR	
Aging Time		Ⅰ + Ⅱ	Ⅲ	Ⅰ + Ⅱ	Ⅲ	Ⅰ + Ⅱ	Ⅲ
0 h		/	79.2	15.7	33.4	16.5	32.2
12 h		/	69.9	18.1	30.9	22.5	20.0
24 h		/	65.6	19.2	27.2	30.8	17.6
36 h		/	61.3	24.4	23.8	31.0	14.8

## Data Availability

Raw data are available upon request.
